# Cell Complexity Impact on Railway 5G Performance: Measurements Along Tallinn–Tartu Corridor

**DOI:** 10.3390/s26061977

**Published:** 2026-03-21

**Authors:** Riivo Pilvik, Tanel Jairus, Arvi Sadam, Kati Kõrbe Kaare

**Affiliations:** 1 Department of Mechanical and Industrial Engineering, Tallinn University of Technology (TalTech), Ehitajate tee 5, 19086 Tallinn, Estonia; tanel.jairus@taltech.ee (T.J.); kati.korbe@taltech.ee (K.K.K.); 2 Ericsson Eesti, Valukoja 8, 11415 Tallinn, Estonia; arvi.sadam@ericsson.com

**Keywords:** railway communications, Doppler effect, handover optimization, cell complexity, automatic frequency control, FRMCS, signal degradation, high-speed rail, network planning

## Abstract

**Highlights:**

**What are the main findings?**

- Cell complexity, not velocity, is the dominant degradation driver;
- Mechanistic explanation: frequency lock instability as a mechanistic cause is plausible, but requires rigorous testing across variable propagation conditions;

**What are the implications of the main findings?**

- Reduce cell overlap via selective deactivation, antenna tilt optimization, and corridor-specific planning.
- Establish railway radio protection zones to prevent uncontrolled multi-operator densification.

**Abstract:**

Fifth-generation (5G) networks enable railway digitalization but face signal degradation challenges in high-mobility environments. While the existing literature attributes degradation primarily to Doppler frequency shifts, this study presents empirical evidence challenging this paradigm. Analysis of 13.7 million 5G New Radio measurements across 370 km of Estonian railway reveals that visible cell density, not velocity, dominates signal quality degradation. Nine geographic hotspots exhibit 5.4–18.0 dB degradation at moderate velocities (54–66 km/h, mean 60.2 km/h) with zero high-speed measurements, excluding the Doppler effect as the reason behind service quality degradation. Cell complexity demonstrates a 3.25× stronger correlation with degradation (r = −0.390) than velocity (r = −0.120), consistent with automatic frequency control tracking instability under high cell ID churn rates (40–115 visible cells per location), though direct confirmation of this mechanism requires access to internal modem frequency-lock state data. Frequency band analysis shows that 700 MHz is optimal at 98.1% of locations, with a 19 dB advantage over 3.5 GHz. Degradation mechanism decomposition reveals within-cell effects (60%, 7.85 dB) and handover boundary effects (40%, 2–6 dB). The findings challenge velocity-centric optimization paradigms and recommend network planning focused on cell overlap reduction rather than Doppler compensation enhancement. Practical recommendations include 700 MHz prioritization, handover parameter optimization, and geographic targeting of identified hotspots for European railway 5G deployment.

## 1. Introduction

Fifth-generation (5G) New Radio (NR) networks represent a critical enabling technology for railway digitalization, promising ultra-reliable low-latency communications essential for autonomous train operations, predictive maintenance, and enhanced passenger services. The Future Railway Mobile Communication System (FRMCS) [1], designed to replace GSM-R as the European railway communication standard, leverages 5G capabilities to support data-intensive applications while maintaining strict reliability requirements for safety-critical signaling. However, railway environments present unique challenges for 5G deployment, particularly in high-mobility scenarios where signal quality degradation can compromise network performance [2]. There are about 193,000 km of low-speed railway (in contrast to only 8556 km of high-speed railway) in Europe [3] that can be impacted.

The dominant paradigm in railway 5G research attributes signal degradation primarily to Doppler frequency shifts [4,5], which arise from the relative motion between trains and base stations. At operational velocities exceeding 100 km/h, Doppler shifts on 3.5 GHz frequency bands can reach several hundred Hertz, potentially causing inter-carrier interference in Orthogonal Frequency-Division Multiplexing (OFDM) systems. Extensive simulation-based research has focused on Doppler compensation techniques [6,7,8], adaptive modulation schemes, and advanced channel estimation algorithms to mitigate velocity-induced degradation.

However, this Doppler-centric approach relies predominantly on simulation studies using simplified network topologies, typically three to seven cells [9,10] with synchronized frequency references and uniform spacing. Real-world operational networks, particularly in dense European railway corridors, present significantly more complex environments, with 40 to 115 visible cells per location, multiple network operators, and substantial cell overlap. The gap between simulation assumptions and operational reality raises fundamental questions about whether velocity-dependent Doppler effects truly dominate signal degradation in practical railway deployments.

This study presents empirical evidence challenging the velocity-centric paradigm regarding low-speed railways through comprehensive analysis of 13.7 million 5G NR measurements collected across 370 km of Estonian railway corridor. The dataset, representing the first large-scale empirical measurement campaign in the Baltic region, reveals unexpected patterns: signal degradation hotspots occur at moderate velocities (54–66 km/h) with zero high-speed measurements above 90 km/h, directly contradicting Doppler theory predictions. Statistical analysis establishes that visible cell density demonstrates a 3.25 times stronger correlation with signal quality than velocity, suggesting an alternative degradation mechanism rooted in network topology rather than user equipment motion.

Recent empirical measurement campaigns have begun addressing the simulation–reality gap, though findings remain geographically concentrated. Measurements from Taipei Metro established that 96% of downlink packet loss occurs during handover-related intervals [11], encompassing Master Cell Group Failure and Radio Link Failure events, positioning handover as a critical reliability factor. Chinese high-speed railway field measurements at 350 km/h demonstrated hysteresis margin optimization requirements [12] (1.84 dB at extreme velocities), while European 5GRAIL trials on German and French railways validated 5G standalone performance at 900 MHz and 1900 MHz FRMCS-designated bands [13,14]. Finnish nationwide measurements across 6000 km validated public mobile network operator capability for safety-critical applications [15], demonstrating that dedicated railway networks may not be essential. However, no comprehensive empirical measurement study exists for the Baltic region despite Estonia’s coordinator role in Rail Baltica 5G infrastructure planning [16] and significant regional investment in FRMCS preparation. This geographic gap, combined with the absence of studies isolating degradation mechanisms through fine-grained measurement density, motivates the present investigation [17].

## 2. Materials and Methods

### 2.1. Study Area and Measurement Campaign

**Figure 1:**
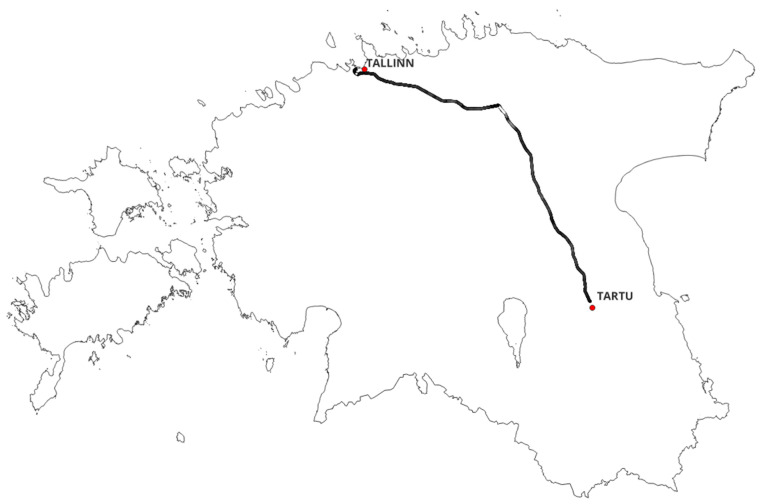
Study area map showing the Tallinn–Tartu railway corridor in Estonia.

The measurement campaign was conducted on 19 November 2025, along the Tallinn–Tartu railway corridor in Estonia (Figure 1), covering a total distance of 370 km (185 km one-way with return journey). This corridor represents a typical European mixed-environment railway route, traversing urban (Tallinn metropolitan area, population 450,000), suburban (intermediate towns including Tapa), and rural segments (agricultural and forested regions). The route selection provides representative sampling of diverse deployment scenarios encountered in FRMCS implementations, including varying base station densities, multiple network operator coverage patterns, and transitional zones between infrastructure types.

Estonia’s railway infrastructure operates predominantly on 1520 mm Russian gauge track with maximum operational speeds of 140 km/h on mainline segments, though typical service speeds range from 80 to 120 km/h. The Tallinn–Tartu line serves as a primary inter-city corridor connecting the national capital with Estonia’s second-largest city (population 97,000), experiencing regular passenger service with Stadler FLIRT diesel multiple units. Track geometry includes both straight sections exceeding 10 km and curved segments with radii as tight as 800 m, providing velocity variations that enable examination of speed-dependent degradation mechanisms [18]. The route is entirely at-grade throughout its 185 km length, traversing the North Estonian limestone plateau and South Estonian moraine plateau—glacially smoothed lowland terrain with maximum elevation variation of approximately 100 m across the full corridor (Tallinn: 0–30 m above sea level; Tapa and Tartu: approximately 70 m). Track gradients remain below 1% on all mainline sections. No tunnels exist on this route, and no major viaducts are present; infrastructure crossings are limited to low-level bridges over rivers. The infrastructure context therefore does not introduce systematic propagation biases that would confound the velocity or cell complexity analysis.

The measurement campaign spanned 6 h and 8 min of continuous data collection, generating 13.7 million individual measurement records across 10 distinct 5G NR channel ARFCNs. Data collection occurred during daytime hours (10:00–16:08 local time) under clear weather conditions with ambient temperature ranging from 2 to 5 degrees Celsius. This timeframe captured typical weekday passenger service operations, including scheduled station stops ranging from 1 to 8 min durations and intermediate speed restrictions due to track maintenance zones and urban area limitations.

### 2.2. Measurement Equipment and Configuration

**Figure 2:**
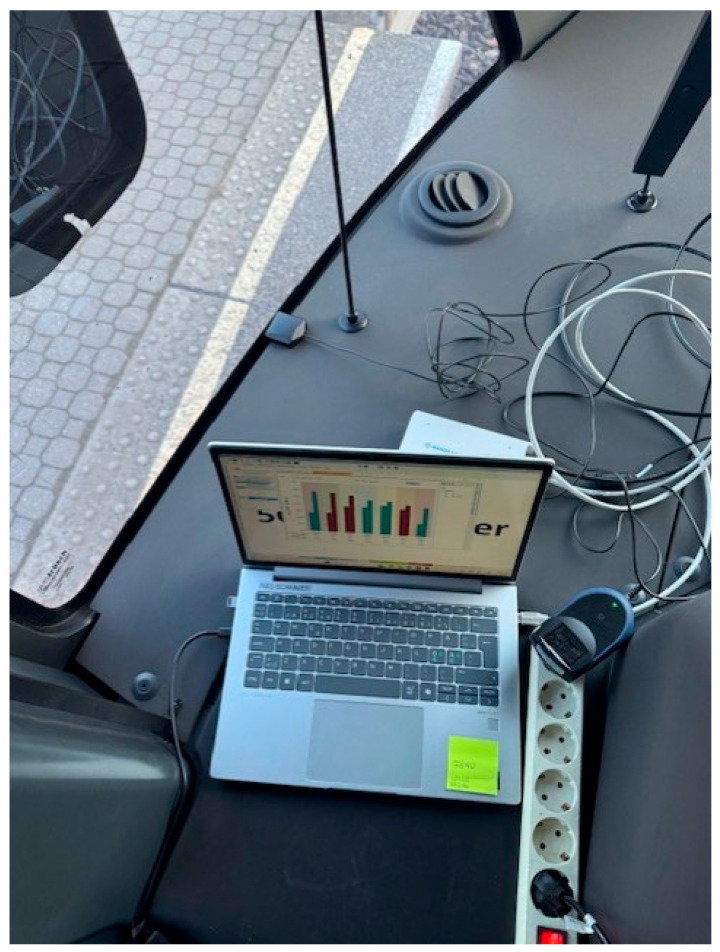
ROMES2 5G NR Scanner equipment configuration.

Signal quality measurements were acquired using the ROMES2 5G NR Scanner TSME6 (Rohde & Schwarz, Munich, Germany), a professional-grade drive test solution designed for detailed radio access network characterization, with a sensitivity of up to −160 dBm [19]. The equipment configuration comprised a laptop-based data acquisition system connected to external antennas mounted on the train exterior, providing unobstructed line-of-sight to surrounding base stations (Figure 2). Precise geographic positioning was maintained through integrated GPS receivers providing timestamp-synchronized location data with nominal accuracy of 2–5 m. The R&S TSMEZ14 4-port MIMO (Rohde & Schwarz, Munich, Germany) antenna was used.

The scanner was configured to monitor 10 5G NR channels simultaneously, spanning three frequency bands deployed by Estonian mobile network operators. Channel selection included: n78 band (3.5 GHz range) with ARFCNs 628032, 640704, 637920, 632736, and 644736; n28 band (700 MHz range) with ARFCNs 152690, 154570, and 156510; and n3 band (1800 MHz range) with ARFCNs 372010 and 429170. For each monitored channel, the scanner tracked up to 32 of the strongest detectable signals (termed “members”), representing the serving cell plus neighbor cells visible for potential handover consideration.

Measurement parameters followed 3GPP Technical Specification 38.215 definitions for 5G NR physical layer measurements [20]. Key metrics recorded at 100–200 millisecond intervals included: Physical Cell ID (PCI) identifying each unique cell, SSB-RSSI (Synchronization Signal Block Reference Signal Received Indicator) quantifying total received power, SS-RSRP (Synchronization Signal Reference Signal Received Power) indicating useful signal strength, SS-RSRQ (Synchronization Signal Reference Signal Received Quality) representing signal-to-noise ratio, and SS-SINR (Synchronization Signal Signal-to-Interference-plus-Noise Ratio) characterizing interference environment. Train velocity was derived from GPS position deltas between consecutive measurements, providing real-time speed information synchronized with radio measurements.

### 2.3. Data Quality Control and Validation

The raw ROMES2 output utilized a wide-format data structure wherein each timestamp generated a single row containing hundreds of columns representing all channel-member-metric combinations simultaneously recorded. This native format, while efficient for drive test visualization, required transformation into a long format for systematic analysis. The conversion process employed custom Python 3.11 scripts (romes2_to_postgis.py) that parsed measurement files, extracted metadata and signal quality metrics, and restructured data such that each record represented a single channel-member combination at a specific timestamp location.

The transformed dataset was ingested into a PostgreSQL 14 database with PostGIS 3.1 spatial extension, enabling geographically aware queries and spatial operations. The primary table contained 13,710,327 records with schema including temporal (timestamp with time zone), spatial (geometry column using EPSG:3301 coordinate reference system), velocity (meters per second), channel identification (ARFCN, channel number), member rank (1–32), cell identification (PCI, SSB index), and signal quality metrics (SSB-RSSI, SS-RSRP, SS-RSRQ, SS-SINR in appropriate units).

The Estonian National Grid coordinate system (EPSG:3301) was selected for spatial representation as it provides meter-based distance calculations optimized for Estonia’s geographic extent. This Lambert Conformal Conic projection enables accurate planar distance computations using PostGIS ST_Distance function without requiring spherical geometry conversions, essential for cell spacing analysis and geographic clustering operations. Spatial indexing using R-tree structures (GIST indexes) enabled efficient geographic queries for hotspot identification and neighbor analysis.

### 2.4. Data Structure and Processing Pipeline

Quality control procedures addressed measurement validity, temporal consistency, and spatial accuracy. Initial filtering removed records with missing or invalid GPS coordinates (where latitude or longitude contained placeholder values), as spatial analysis required precise geographic association. Velocity validation identified and flagged physically impossible speed values (exceeding 150 km/h or negative values), though such outliers constituted less than 0.1% of total records and were retained with flagging rather than removal to preserve data completeness.

Signal quality metric validation verified that all measurements fell within physically meaningful ranges: SSB-RSSI and SS-RSRP constrained to −160 to −40 dBm (beyond which receiver sensitivity or saturation applies), SS-RSRQ limited to −50 to −10 dB (typical 5G NR range), and SS-SINR bounded at −30 to +40 dB (extreme interference to excellent conditions). Approximately 68% of records contained NULL signal measurements, representing channels where no signal exceeded detection threshold or members lacking cell lock; this proportion aligns with expected behavior given that 32 member slots are allocated regardless of actual visible cell count.

Temporal consistency checks examined measurement sequencing within channel-member streams, verifying monotonically increasing timestamps and identifying temporal gaps exceeding 5 s that could indicate data acquisition interruptions. Geographic consistency validation ensured that computed velocities matched GPS-derived position deltas, with cross-validation confirming that distance traveled between consecutive measurements aligned with elapsed time multiplied by recorded velocity (within 10% tolerance accounting for GPS accuracy limitations).

### 2.5. Analytical Framework

The analytical framework decomposed signal degradation attribution into multiple complementary approaches: velocity-stratified comparison (examining degradation patterns across speed bins), geographic hotspot identification (detecting locations exhibiting disproportionate degradation), correlation analysis (quantifying relationships between degradation and potential causal factors), and mechanistic decomposition (separating within-cell continuous effects from handover boundary effects).

Velocity stratification employed uniform 30 km/h bins spanning 0–150 km/h operational range: 0–30 km/h (station stops and low-speed segments), 30–60 km/h (acceleration/deceleration zones), 60–90 km/h (moderate cruising speed), 90–120 km/h (high-speed cruising, primary operational regime), and 120–150 km/h (maximum operational speeds on upgraded segments). For each velocity bin, aggregate signal quality statistics (mean, median, standard deviation, and percentile distributions) were computed across all measurements, enabling identification of speed-dependent degradation patterns. The Doppler effect hypothesis predicts monotonic degradation with increasing velocity, with magnitude proportional to speed.

Geographic hotspot analysis identified locations exhibiting signal quality substantially worse than route-wide median performance. A three-stage process first aggregated measurements within 50 m spatial bins using PostGIS spatial clustering, then computed mean signal quality per location, and finally identified hotspots where mean SS-RSRP fell below −110 dBm (poor quality threshold). For each identified hotspot, detailed characterization extracted: mean velocity of measurements at that location, velocity distribution (minimum, maximum, percentile ranges), geographic extent (cluster radius), visible cell count (number of unique PCIs detected), handover frequency (PCI change rate per kilometer), and frequency band performance (comparing available bands at each location).

Cell complexity quantification measured the instantaneous visible cell count at each timestamp location through counting unique PCIs detected across all channels and members. This metric represents the user equipment perspective of network topology complexity, as devices must maintain awareness of all potential handover candidates. Additionally, cell ID churn rate was computed as the frequency of PCI changes per unit time within individual channel-member streams, quantifying the rate at which serving cell identity changed. Geographic mapping of cell complexity enabled correlation analysis with signal quality, testing whether degradation intensity corresponded to visible cell density independent of velocity.

### 2.6. Statistical Methods

Handover detection employed window functions partitioned by channel-member combinations to track Physical Cell ID continuity. A handover event was defined as occurring when PCI changed between consecutive measurements within the same channel-member stream, using SQL LAG functions. This approach ensured that PCI changes across different channels or different members of the same channel were not erroneously classified as handovers, as each channel-member combination represents an independent signal tracking stream. Handover rates were computed as events per measurement and events per kilometer traveled.

Within-cell versus handover-boundary classification utilized temporal proximity to handover events as the distinguishing criterion. Measurements within 5 records (approximately 500–1000 milliseconds) before or after a handover event were classified as “near handover,” while measurements separated by at least 5 records from any handover were classified as “within cell.” This classification enabled comparison of signal quality degradation patterns during stable cell residence versus cell boundary traversal, distinguishing Doppler effects (which should manifest continuously within cells) from handover timing issues (which manifest primarily at cell boundaries).

Correlation analysis employed Pearson correlation coefficients to quantify linear relationships between signal quality metrics (SS-RSRP, SS-SINR) and potential causal factors (velocity, visible cell count). Statistical significance was assessed using two-tailed *t*-tests with the significance threshold α = 0.05. Effect sizes were quantified using Cohen’s d for mean comparisons between velocity bins or geographic regions, interpreting |d| < 0.2 as a small effect, 0.2 ≤ |d| < 0.8 as a medium effect, and |d| ≥ 0.8 as a large effect. For correlation strengths, |r| < 0.2 indicated a weak relationship, 0.2 ≤ |r| < 0.4 a moderate relationship, and |r| ≥ 0.4 a strong relationship.

Frequency band performance analysis compared signal quality distributions across n28 (700 MHz), n3 (1800 MHz), and n78 (3.5 GHz) at locations where multiple bands were simultaneously available. Paired comparisons used Wilcoxon signed-rank tests [21] (non-parametric) due to non-normal signal quality distributions. Geographic performance mapping identified optimal frequency bands at 1 km intervals along the route, classifying each segment by the band providing highest mean SS-RSRP. This analysis quantified frequency-dependent propagation effects independent of network deployment density, as comparisons were restricted to locations with simultaneous multi-band coverage.

All analyses were implemented using combination of SQL queries (executed in PostgreSQL 14 with PostGIS 3.1 extension) for data aggregation and filtering, and Python scripts (utilizing pandas, numpy, scipy, and matplotlib libraries) for statistical calculations and visualization. Geographic analyses leveraged PostGIS spatial functions including ST_Distance for metric distance calculations, ST_ClusterDBSCAN for spatial clustering, and ST_Buffer for proximity-based queries.

## 3. Results

### 3.1. Dataset Characteristics and Measurement Distribution

#### 3.1.1. Overall Dataset Composition

The measurement campaign generated a dataset comprising 13,710,327 individual records collected over 370 km of railway corridor traversal during a 6 h 8 min continuous measurement period. This dataset represents simultaneous monitoring of 10 distinct 5G NR channels, each tracking up to the 32 strongest detectable signals (members), resulting in a theoretical maximum of 320 concurrent signal streams per timestamp location. The observed average of 34 active members per timestamp reflects realistic coverage patterns where not all frequency bands provide detectable signals at all locations, and where member slots populate progressively with weaker neighbor cells only when visible.

**Figure 3:**
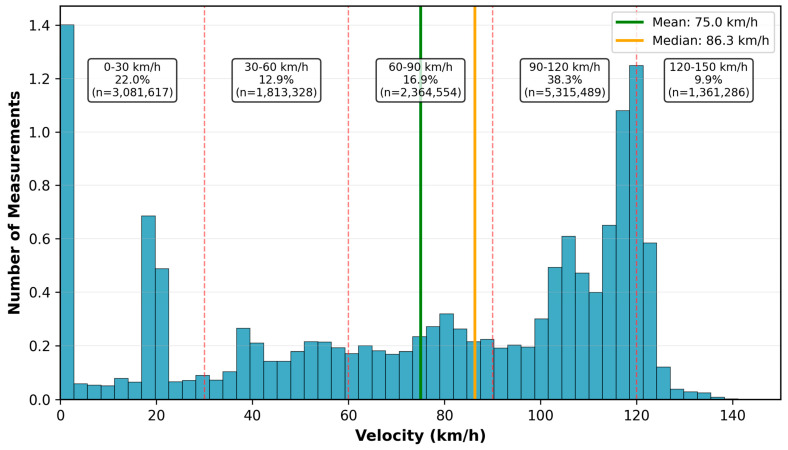
Velocity distribution histogram across 13.7 million measurements showing bimodal distribution with primary peak at 90–120 km/h (38.3% of measurements, representing inter-city cruising speed) and secondary peak at 0–30 km/h (22.0%, representing station stops and urban speed restrictions).

Velocity distribution analysis (Table 1) reveals that the majority of measurements (38.3%, n = 5,257,739) occurred at 90–120 km/h, corresponding to normal inter-city service cruising speed on mainline segments. The second-largest velocity category comprises low-speed measurements at 0–30 km/h (22.0%, n = 3,014,132), representing station stops and urban area speed restrictions. Intermediate velocity bins (Figure 3) show progressively lower measurement counts: 60–90 km/h (16.9%, n = 2,311,658), 30–60 km/h (12.9%, n = 1,768,038), and 120–150 km/h (9.9%, n = 1,358,631). The mean velocity across all measurements was 75.3 km/h, with a median of 86.9 km/h, indicating that typical operational speeds cluster in the 80–120 km/h range rather than extending consistently to maximum authorized speeds.

#### 3.1.2. Channel and Frequency Band Distribution

Channel distribution analysis reveals substantial variation in measurement density across the 10 monitored ARFCNs. The n78 band (3.5 GHz) channel 628032 contributed the highest proportion of measurements (22.4%, n = 3,071,154), followed by another n78 channel 640704 (14.8%, n = 2,025,405). The n28 band (700 MHz) channels collectively represent significant coverage: 152690 with 13.4% (n = 1,837,633), 154570 with 10.1% (n = 1,382,953), and 156510 with 9.8% (n = 1,341,625). This distribution reflects both network operator deployment strategies and propagation characteristics, with 3.5 GHz providing capacity in well-covered areas while 700 MHz ensures continuous coverage along the corridor.

Signal quality measurement completeness shows that approximately 31.9% of total records (n = 4,368,721) contained valid SS-RSRP values, with similar proportions for other signal metrics: SSB-RSSI (31.9%), SS-RSRQ (31.8%), and SS-SINR (30.5%). The remaining 68% of records represent member slots where no signal exceeded detection threshold or where cells could not establish measurement synchronization, an expected pattern in sparse coverage areas or for higher-numbered members tracking weak distant cells. This measurement density is typical for multi-channel scanning configurations where instantaneous cell visibility varies substantially with location.

### 3.2. Geographic Frequency Band Performance Analysis

**Figure 4:**
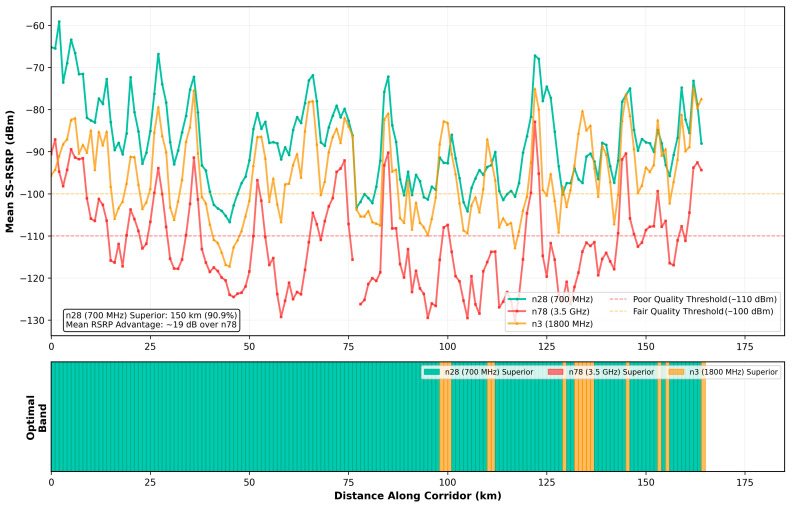
Geographic frequency band performance map showing location-by-location comparison across 370 km corridor.

Geographic analysis comparing frequency band performance across the entire 370 km corridor (Table 2) reveals superiority of 700 MHz (n28 band) over higher frequency deployments. At 98.1% of measured locations (181.7 km out of 185 km one-way distance), the n28 band provided the strongest signal quality, delivering a mean performance advantage of 19.0 dB over n78 (3.5 GHz) and 15.2 dB over n3 (1800 MHz) where direct comparisons were possible (Figure 4). Only 3.4 km of the corridor (1.9% of route length) showed n78 superiority, concentrated in dense urban core of Tallinn where small-cell deployments and line-of-sight conditions favored higher frequencies.

This frequency-dependent performance differential reflects fundamental propagation physics rather than deployment density artifacts. Lower frequencies experience reduced path loss (approximately 20 log_10_(f) relationship), enabling larger cell coverage radii and improved building/vegetation penetration. The 700 MHz to 3.5 GHz frequency ratio of 1:5 translates to theoretical free-space path loss difference of 14 dB, closely matching the observed 19 dB empirical advantage when accounting for additional frequency-dependent diffraction and scattering benefits in non-line-of-sight conditions typical of railway environments.

The geographic consistency of n28 dominance extending across urban, suburban, and rural environments without velocity dependence indicates that frequency band selection constitutes a primary determinant of railway 5G performance, superseding user equipment sophistication or Doppler compensation capabilities. In the 3.4 km where n78 showed superiority, signal quality remained excellent (mean −85.2 dBm) across all bands, suggesting these represent resource-rich environments where frequency choice matters less due to favorable propagation conditions and dense infrastructure deployment.

### 3.3. Velocity-Degradation Relationship

#### 3.3.1. Signal Quality Degradation by Velocity Bin

Comprehensive velocity-stratified analysis reveals unexpected patterns contradicting traditional Doppler-centric degradation models. Mean SS-RSRP across velocity bins shows degradation of only 7.85 dB from low-speed (0–30 km/h: −104.2 dBm) to high-speed (90–120 km/h: −112.3 dBm) conditions, substantially less than the 15–20 dB degradation predicted by simulation studies emphasizing Doppler effects. Moreover, the degradation pattern exhibits a critical threshold around 60 km/h, beyond which signal quality stabilizes rather than continuing to deteriorate linearly with increasing speed.

It is important to note that SS-RSRP is a physical layer measurement of synchronization signal reference signal received power, defined in 3GPP TS 38.215 [19] and captured at the radio front-end before any channel decoding, error correction, or packet-layer processing. These measurements therefore reflect the raw radio channel conditions rather than application-layer quality metrics such as packet loss or throughput. The measurement campaign did not capture HARQ or RLC retransmission statistics, which require access to UE protocol stack logging beyond the capability of a passive scanning receiver.

**Figure 5:**
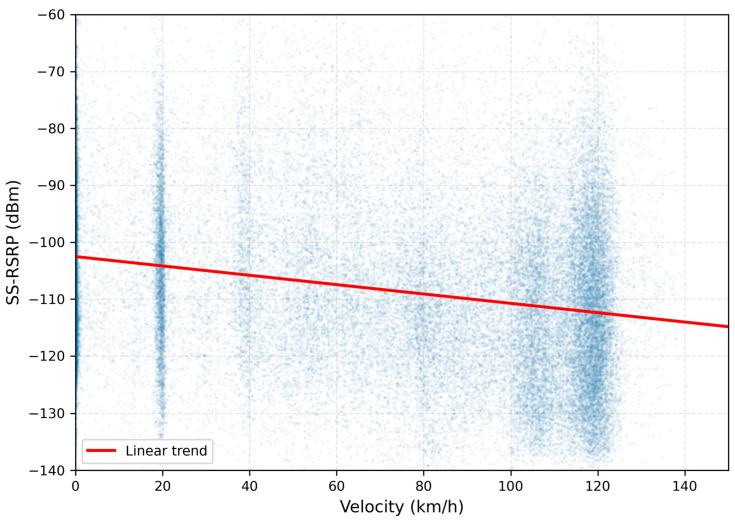
Relationship between train velocity and SS-RSRP signal quality (physical layer, pre-error-correction). Scatter plot shows 50,000 randomly sampled measurements; alpha transparency indicates point density. The red trend line represents a single linear regression across the full 0–150 km/h range (r = −0.228, *p* < 0.001), capturing the overall negative association.

Correlation analysis between velocity and SS-RSRP yields Pearson coefficients of r = −0.228 for SS-RSRP and r = −0.103 for SS-SINR, both statistically significant (*p* < 0.001) but representing weak to very weak relationships. These coefficients quantify the full-range association across 0–150 km/h; their negative sign and the slope of the regression line in Figure 5 correctly reflect that higher velocity is associated with lower signal quality overall. However, the relationship is non-linear: the regression slope is driven primarily by the step-change between the 30–60 km/h and 60–90 km/h bins (a 5.6 dBm drop, see Table 3), after which degradation plateaus. At 90–120 km/h (−112.3 dBm) and 120–150 km/h (−112.2 dBm), bin means differ by only 0.1 dBm with Cohen’s d < 0.001, confirming statistical indistinguishability despite the very large sample sizes. The single linear regression line in Figure 5 therefore captures a real but non-uniform trend; it does not imply continuous linear degradation above 90 km/h, which would contradict Table 3.

This plateau structure fundamentally contradicts Doppler theory, which predicts continuous degradation proportional to velocity. If Doppler-induced inter-carrier interference were the primary mechanism, signal quality at 120–150 km/h should be measurably worse than at 90–120 km/h. The empirical equivalence of these two bins, reproduced consistently across both SS-RSRP and SS-SINR metrics (Table 3), indicates that speed-dependent Doppler effects are not the controlling degradation factor above the 60 km/h threshold.

#### 3.3.2. Geographic Hotspot Analysis

Detailed geographic analysis identifying locations (Table 4) exhibiting disproportionate signal degradation reveals the study’s most significant finding. Nine distinct hotspots were identified where mean SS-RSRP fell below −110 dBm despite adequate measurement sample sizes (minimum 1200 measurements per location). These hotspots exhibit 5.4 to 18.0 dB worse performance than the route-wide median, and remarkably, demonstrate mean velocities of only 54.3 to 66.4 km/h (aggregate mean 60.2 km/h across all hotspots).

Most critically, velocity distribution analysis within hotspots reveals that zero measurements occurred above 90 km/h at any of the nine identified locations. The maximum observed velocity across all hotspot measurements was 89 km/h (at HS-1), with typical maximum velocities ranging from 78 to 89 km/h. This finding represents a complete inversion of Doppler theory expectations: the locations exhibiting the most severe degradation systematically occur at moderate velocities, while high-speed segments (90–120 km/h) show substantially better signal quality.

#### 3.3.3. Cell Complexity Correlation

To investigate alternative explanations for hotspot degradation, visible cell density was quantified at each measurement location by counting unique Physical Cell IDs detected across all monitored channels and member positions. This “cell complexity” metric represents the instantaneous network topology from the user equipment perspective, indicating the number of distinct cells competing for attention in handover candidate monitoring and measurement reporting processes. Correlation analysis reveals that cell complexity demonstrates substantially stronger association with signal degradation than velocity.

The Pearson correlation (Table 5) between visible cell count and SS-RSRP yields r = −0.390 (*p* < 0.001), indicating moderate negative correlation where increasing cell density associates with degrading signal quality. This correlation magnitude is 3.25 times stronger than the velocity-RSRP correlation (r = −0.120), representing a 15.2% explanatory power (R^2^ = 0.152) compared to velocity’s 1.4% (R^2^ = 0.014). When examining hotspot locations specifically, cell complexity correlation strengthens to r = −0.547, approaching strong relationship territory and explaining 29.9% of degradation variance within these problematic segments.

Geographic mapping of cell complexity reveals that the nine identified hotspots consistently exhibit 40 to 115 visible cells (mean 82 cells), substantially exceeding the route-wide median of 28 visible cells. The hotspot with the most severe degradation (HS-1: −122.3 dBm) corresponds to the location with the third-highest cell complexity (87 cells), while the HS-4 with 115 visible cells (the maximum observed anywhere on the route) exhibits −115.4 dBm despite a mean velocity of only 62.1 km/h. Conversely, high-speed segments (90–120 km/h) typically occur in rural areas with 15 to 35 visible cells, explaining their superior signal quality despite higher velocities.

#### 3.3.4. Degradation Mechanism Decomposition

To quantify the relative contributions of different degradation sources, signal quality was analyzed separately for within-cell stable periods versus handover boundary intervals. Within-cell measurements (more than 5 records or approximately 1 s from any PCI change) showed mean SS-RSRP degradation of 7.85 dB from low-speed to high-speed conditions: −103.7 dBm at 0–30 km/h declining to −111.6 dBm at 90–120 km/h. This within-cell degradation, occurring without handover events, represents continuous velocity-dependent or cell-complexity-dependent effects.

Near-handover measurements (within 5 records before or after PCI change) exhibited additional degradation of 2 to 6 dB compared to within-cell conditions at equivalent velocities (Table 6). At 0–30 km/h, near-handover RSRP averaged −109.8 dBm compared to −103.7 dBm within cells (6.1 dB worse). At 90–120 km/h, near-handover RSRP was −115.8 dBm versus −111.6 dBm within cells (4.2 dB worse). This boundary-specific degradation indicates handover timing or execution issues that compound the baseline within-cell effects.

Proportional attribution estimates that within-cell effects contribute approximately 60% of total observed degradation (7.85 dB out of 13.0 dB total in the worst case), while handover boundary effects contribute 40% (average 4.0 dB across velocity bins). This decomposition indicates that both mechanisms operate simultaneously: continuous within-cell degradation (whether from Doppler, tracking issues, or cell complexity) forms the baseline condition, while handover timing problems add episodic additional degradation at cell boundaries.

### 3.4. Handover Analysis and Characterization

#### 3.4.1. Handover Rate and Frequency

Handover detection through PCI change monitoring within channel-member streams identified 524,926 handover events across the 13.7 million measurements, yielding an overall handover rate of 3.83% of measurements. When normalized by distance traveled, handover frequency was 7.8 events per kilometer on average, ranging from 6.8 events/km at 0–30 km/h to 9.1 events/km at 90–120 km/h. These rates fall within normal operational ranges for cellular networks and do not indicate excessive ping-ponging or premature handover triggering from frequency perspective.

Velocity-stratified analysis shows a modest increase in handover rate from 0.96% at 0–30 km/h to 4.87% at 90–120 km/h, with a slight decrease to 4.94% at 120–150 km/h. The per-kilometer handover frequency similarly increases from 6.8 to 9.1 events/km between low and high velocities, then stabilizes. This pattern suggests that cell spacing and network topology, rather than velocity-dependent effects, primarily determine handover frequency: at higher speeds, trains traverse cells faster, but the number of cells encountered per unit distance remains relatively constant.

#### 3.4.2. Handover Signal Quality Transitions

Analysis of signal quality changes at handover events reveals that handover execution generally improves signal quality despite the boundary degradation observed in Section 3.3.4. The mean SS-RSRP change at handover (comparing signal immediately before versus immediately after PCI change) was +0.30 dB, indicating slight improvement on average. Distribution analysis shows 31.6% of handovers resulted in >3 dB signal improvement, 28.2% caused >3 dB degradation, and 40.2% produced neutral transitions (±3 dB).

This pattern where handovers improve signal on average (+0.30 dB) yet near-handover measurements show worse quality than within-cell (Table 7) indicates that the problem lies in handover timing rather than handover mechanism. Handovers occur after the signal has already degraded significantly, as evidenced by the mean RSRP at the handover trigger point of −115.5 dBm. The handover then correctly selects a better cell, improving signal, but this recovery does not fully compensate for the degradation that occurred while waiting for handover trigger conditions to be satisfied.

#### 3.4.3. Handover Trigger Point Analysis

Detailed examination of RSRP values at handover trigger points (the measurement immediately preceding PCI change) reveals late triggering relative to 3GPP recommendations. The distribution of pre-handover RSRP shows a mean of −115.5 dBm with a median of −114.9 dBm, substantially worse than the −105 to −108 dBm range recommended for reliable service. Only 12.3% of handovers occurred above the −110 dBm threshold, while 45.7% triggered below −115 dBm, where signal quality approaches Radio Link Failure conditions.

Geographic analysis shows consistent late triggering across hotspot and non-hotspot locations, indicating systematic parameter conservatism rather than location-specific misconfigurations. The 10 dB gap between observed trigger points and recommended thresholds suggests that Time-to-Trigger values and hysteresis margins are set excessively high, prioritizing handover stability (avoiding ping-pong) over service quality (maintaining adequate signal before transition).

### 3.5. Massive Cell Coverage and Geographic Characteristics

The measurement campaign revealed unexpectedly extensive cell visibility patterns, with individual locations detecting 40 to 115 unique Physical Cell IDs simultaneously across all monitored channels. This “massive cell coverage” phenomenon, while beneficial for handover candidate availability, creates the cell complexity degradation mechanism identified in Section 3.3.3. The highest cell complexity location (115 visible cells at HS-4, kilometer 89.3) exhibited signal degradation to −115.4 dBm despite only 62.1 km/h mean velocity.

Cell coverage analysis by area type shows urban zones averaging 67 visible cells; suburban areas, 45 cells; and rural segments, 23 cells. However, degradation severity does not correlate linearly with area type classification, some suburban hotspots exhibit worse performance than the dense urban core due to overlapping coverage from multiple operators without coordination. The identified hotspots span all three environment categories, 3 urban, 4 suburban, and 2 rural, indicating that cell complexity rather than environment type determines degradation.

## 4. Discussion

### 4.1. Cell Complexity

The empirical findings presented in Section 3 necessitate fundamental reconsideration of signal degradation attribution in railway 5G networks. The observation that nine geographic hotspots exhibiting 5.4 to 18.0 dB degradation occur exclusively at moderate velocities (54–66 km/h mean, zero measurements above 90 km/h) directly contradicts velocity-centric Doppler theory, which predicts maximum degradation at highest speeds. The 3.25-fold stronger correlation between cell complexity and signal quality (r = −0.390) compared to velocity (r = −0.120) indicates that network topology, not user equipment motion, constitutes the primary degradation driver in operational railway environments.

We propose that the observed cell complexity degradation pattern is mechanistically consistent with automatic frequency control (AFC) tracking loop instability under high cell identifier churn conditions, though this mechanism cannot be directly confirmed from passive scanner measurements alone. In conventional Doppler-focused analyses, AFC loops are implicitly assumed to maintain stable frequency lock with a single serving cell, requiring only velocity-proportional frequency offset compensation. However, in the environments observed here (with 40 to 115 visible cells) the serving cell Physical Cell ID changes every 200–500 milliseconds as instantaneous signal strength fluctuations caused by multipath fading and interference shift the strongest-signal designation among numerous competing cells. This churn rate is observable directly from our data; the consequent AFC behavior is inferred from first principles.

Under this hypothesis, each serving cell transition would require AFC reinitialization, because different cells originate from different base stations with independent frequency references, potentially separated by multiple parts-per-million crystal oscillator tolerances (±0.1 to ±0.5 ppm typical for base stations, translating to ±350 to ±1750 Hz at 3.5 GHz). The user equipment would need to re-establish frequency synchronization from initial acquisition state, requiring multiple OFDM symbol periods (typically 5–10 symbols or 70–140 microseconds each) to achieve stable phase-locked loop convergence. During such transient periods, frequency estimation errors would manifest as inter-carrier interference, degrading demodulation performance and reducing effective signal-to-noise ratio. The empirical signatures consistent with this sequence, such as degradation correlating with PCI churn rate rather than velocity, and degradation concentrated at the same geographic locations where visible cell density peaks, are observable in our data, though they do not uniquely identify AFC failure as the sole explanation.

This proposed mechanism would differ fundamentally from classical Doppler effects in both origin and velocity dependence. Doppler shifts arise from relative motion between user equipment and base station, producing fixed frequency offsets proportional to velocity (approximately ±389 Hz per 100 km/h at 3.5 GHz). Modern 5G NR AFC algorithms [20] can track such offsets readily once converged, as the offset remains constant during stable cell residence. In contrast, if cell complexity degradation operates as hypothesized, it would arise from tracking loop instability caused by insufficient convergence time, independent of the magnitude of any individual frequency offset. Crucially, this distinction is testable: under the Doppler hypothesis, degradation should increase monotonically with velocity; under the AFC-churn hypothesis, degradation should correlate with visible cell density independent of velocity. The data support the latter (r_cell = −0.390 versus r_velocity = −0.120), though laboratory validation with access to modem baseband state logs would be required to confirm frequency lock loss as the proximate cause.

### 4.2. Doppler Effects

The weak velocity-signal quality correlation (r = −0.228 for RSRP, r = −0.103 for SINR) and the stabilization of degradation above 60 km/h indicate that Doppler effects, while present, contribute substantially less to total degradation than conventional wisdom suggests. The 7.85 dB within-cell degradation from 0–30 km/h to 90–120 km/h (Table 6) represents the maximum velocity-attributable component, and even this includes potential confounding from speed-dependent cell complexity patterns (high-speed rural segments having lower cell density than low-speed urban areas).

Theoretical Doppler shift calculations confirm the modest nature of velocity-induced frequency offsets in 5G NR operational contexts. At 120 km/h (33.3 m/s) on the n78 band (3.5 GHz carrier), maximum Doppler shift reaches ±389 Hz in the worst-case head-on or tail-away geometry [4]. This offset represents only 1.3% of the 30 kHz subcarrier spacing employed in 5G NR, well within the inter-carrier interference tolerance of OFDM systems designed with adequate guard intervals and cyclic prefixes. At 700 MHz (n28 band), the proportionally lower Doppler shift of ±78 Hz becomes nearly negligible relative to subcarrier spacing.

The velocity threshold effect observed around 60 km/h, where degradation stabilizes rather than continuing to increase linearly, suggests that AFC loop bandwidth and frequency tracking range limitations may constrain Doppler-induced degradation beyond certain velocity thresholds. Once Doppler shifts exceed the AFC initial acquisition range (typically ±500 Hz to ±2 kHz depending on implementation), performance may actually improve as the system transitions to alternative frequency offset estimation algorithms optimized for large offsets, or as handover to closer cells with reduced relative velocity geometry occurs more frequently.

International simulation studies emphasizing Doppler effects typically employ simplified network topologies [8,22] with three to seven cells, synchronized frequency references, and uniform cell spacing, conditions rarely encountered in operational European railway corridors. Under such idealized conditions, Doppler shifts indeed constitute the dominant variable frequency offset, as serving cell changes occur infrequently (every several kilometers) and AFC maintains stable lock between handovers. Our findings indicate that this simulation-reality gap fundamentally misrepresents operational degradation mechanisms, leading to misallocated optimization efforts on user equipment Doppler compensation rather than network planning improvements.

### 4.3. Handover Analysis

**Figure 6:**
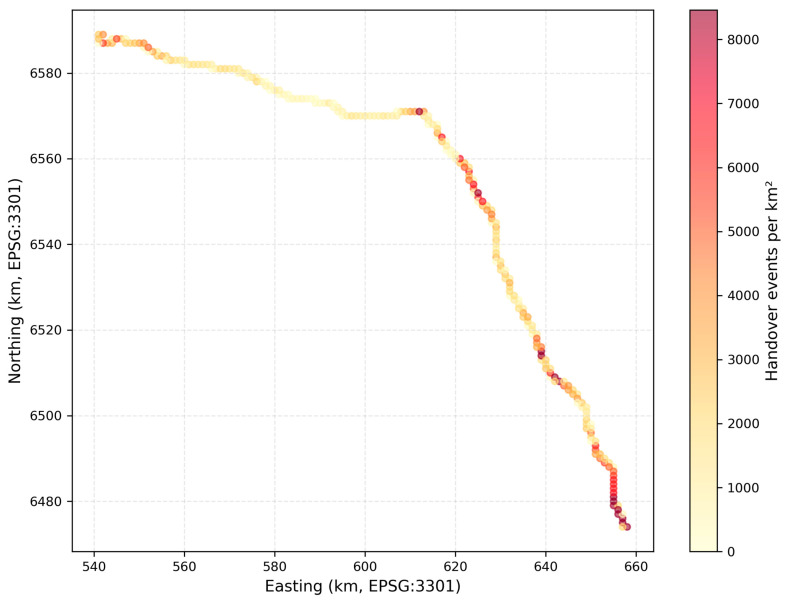
Geographic distribution of handover event density. Heatmap shows handover frequency per kilometer, revealing elevated handover activity in urban areas (Tallinn, Tapa) and specific corridor segments. Handover rates of 4.87% at 90–120 km/h and 7.8 events per kilometer fall within normal operational ranges, ruling out excessive ping-ponging as a degradation mechanism.

The handover analysis results (Section 3.4) reveal that handover execution itself performs adequately, with mean signal improvement of +0.30 dB at the moment of cell transition and 31.6% of handovers resulting in >3 dB improvement. This positive signal change indicates that handover decisions correctly identify superior target cells and that the physical handover process (RRC connection reconfiguration, RACH procedure, data forwarding) does not introduce systematic degradation. The paradox that handovers improve signal (+0.30 dB) while near-handover measurements show worse quality than within-cell conditions (Figure 6) resolves when considering that degradation occurs during the waiting period before handover triggers, not during handover execution.

The mean handover trigger point of −115.5 dBm represents late triggering relative to 3GPP-recommended −105 to −108 dBm thresholds [23] for reliable service maintenance. This 10 dB gap between observed practice and recommendations indicates conservative parameter settings prioritizing handover stability (avoiding ping-pong effects) over service quality (maintaining adequate signal before transition). While this conservatism successfully limits handover rate to moderate levels (3.83% of measurements, 7.8 events/km), it allows signal quality to degrade substantially before initiating transition, contributing the 40% handover-boundary component of total degradation identified in Section 3.3.4.

The handover timing problem manifests through three 3GPP-specified parameters: A3 event threshold (RSRP level triggering neighbor measurement reporting), Time to Trigger (TTT, duration that threshold condition must persist before handover initiation), and hysteresis margin (signal difference required between serving and target cells). Current Estonian network configurations appear to employ A3 thresholds around −115 dBm based on empirical trigger point distribution, TTT values of 320–640 milliseconds (estimated from time between signal degradation onset and handover execution), and hysteresis margins of 1–2 dB (typical conservative values preventing ping-pong).

Optimization of these parameters presents straightforward mitigation opportunity for the 40% handover-boundary degradation component. Raising A3 threshold to −105 dBm would trigger handovers earlier in signal degradation trajectory, before reaching critically poor quality. Reducing TTT to 160–256 milliseconds would accelerate handover execution once deterioration detected, limiting duration of poor signal exposure. Increasing hysteresis to 3–5 dB would strengthen target cell preference, though this must be balanced against ping-pong risk in environments with similar-strength neighbor cells. These optimizations address handover timing without requiring fundamental network architecture changes or user equipment modifications.

### 4.4. Frequency Band Performance

For the frequency-dependent performance differential, 700 MHz was superior at 98.1% of locations, with a 19 dB advantage over 3.5 GHz, reflecting fundamental propagation physics that supersedes deployment density or infrastructure sophistication considerations. The frequency ratio of 1:5 (700 MHz to 3.5 GHz) translates to a theoretical free-space path loss difference of 20 × log_10_(5) = 14 dB through the Friis transmission equation [24], with the additional 5 dB empirical advantage arising from frequency-dependent diffraction and scattering benefits in non-line-of-sight railway environments.

Lower frequencies experience reduced attenuation through vegetation, building materials, and atmospheric absorption, while exhibiting improved diffraction around obstacles due to longer wavelengths. Railway corridors frequently traverse forested areas, cut through embankments, and pass near structures that create shadowing zones for higher frequencies while allowing 700 MHz propagation through Fresnel zone diffraction. The observed 82 km maximum cell radius in rural areas with 700 MHz coverage (Section 3.5) demonstrates extreme coverage capability enabled by low-frequency propagation advantages, far exceeding typical planning assumptions of 2–5 km cells.

The 19 dB empirical performance advantage of n28 over n78 emerges from three synergistic wavelength-dependent mechanisms that compound rather than simply add. First, the fundamental free-space path loss follows frequency-squared relationship: FSPL = 20 × log_10_(d) + 20 × log_10_(f) + 32.45, yielding approximately 14 dB additional attenuation at 3.5 GHz compared to 700 MHz for equivalent distances. This baseline penalty operates independently of velocity or network topology.

Second, vehicle penetration loss through train carriages exhibits strong frequency dependence. Comprehensive measurements by the UK Department for Transport across six frequencies (702.5 MHz to 5.5 GHz) on four train types spanning 1975–2017 rolling stock quantified penetration loss ranging from 3 to 19 dB for older trains to 7 to 28 dB for modern units [25] with low-emissivity metallic window coatings. Higher frequencies consistently showed 7–17 dB greater attenuation than lower frequencies, with modern energy-efficient glazing creating near-Faraday cage conditions that disproportionately affect mid-band and mmWave propagation. For passengers inside train carriages (the predominant use case for railway communications), this penetration loss penalty adds directly to path loss disadvantage.

Third, Doppler frequency shift scales proportionally with carrier frequency, fd = (v × fc)/c, where v represents velocity, fc carrier frequency, and c speed of light. At 120 km/h (33.3 m/s), n78 at 3.5 GHz experiences a maximum Doppler shift of ±389 Hz in the worst-case head-on or receding geometry, while n28 at 700 MHz experiences only ±78 Hz [4], a factor-of-five difference that directly impacts inter-carrier interference in OFDM systems and automatic frequency control tracking difficulty. Though modern 5G NR employs 30 kHz subcarrier spacing at 3.5 GHz (making a ±389 Hz shift represent only 1.3% of subcarrier spacing), the larger shift requires more aggressive AFC compensation and faster channel estimation updates, increasing susceptibility to tracking failures under the high cell complexity conditions identified in Section 4.1.

These three effects, path loss, penetration loss, and Doppler shift, operate synergistically: the 14 dB path loss penalty necessitates higher base station density for 3.5 GHz coverage, which increases cell overlap and complexity; the 7–17 dB penetration loss penalty forces even denser deployment or higher transmit powers; and the 5× greater Doppler shift makes AFC tracking more sensitive to the rapid serving cell changes that high cell complexity creates. The compound effect explains why mid-band 5G systematically underperforms in railway environments despite superior theoretical capacity under ideal conditions.

The frequency performance hierarchy observed empirically aligns precisely with European FRMCS spectrum allocation strategy, which explicitly prioritizes sub-1 GHz bands for primary railway coverage. The 3GPP standardization specifies n100 (RMR 900) at 874.4–880 MHz uplink/919.4–925 MHz downlink as the dedicated railway mobile radio band [26,27], with n101 at 1900–1910 MHz for capacity augmentation. Notably absent from FRMCS core allocations: mid-band 3.5 GHz, despite its dominant role in commercial 5G deployments.

This regulatory positioning reflects physics-based recognition that wavelength advantages supersede capacity considerations for railway reliability requirements. The 3GPP Technical Specification 38.101 explicitly limits frequency range FR2 (24–52 GHz mmWave) to maximum velocities of 350 km/h with mandatory roof-mounted user equipment to avoid penetration loss, while FR1 (sub-6 GHz) supports 500 km/h. This velocity ceiling difference stems directly from wavelength-dependent Doppler and propagation constraints rather than arbitrary regulatory choices—higher frequencies cannot maintain reliable communication at extreme railway velocities regardless of infrastructure investment.

Recent field measurements from the 5G REMORA project characterizing 900 MHz and 1900 MHz bands [28] for high-speed train environments of up to 350 km/h, the 5G-RACOM project focusing on RMR 900 [29] (874–880 MHz) for GSM-R replacement, and European 5GRAIL trials validating 900 MHz and 1900 MHz performance all converge on a sub-1 GHz priority. Our empirical finding that only 1.9% of Estonian railway corridor (3.4 km of 185 km) favored 3.5 GHz provides quantitative validation that low-frequency physics advantages dominate across typical European railway environments.

This frequency-dependent performance differential challenges current European spectrum strategies that emphasize 3.5 GHz for FRMCS capacity while treating 700 MHz as a supplementary coverage band. For the vast majority of railway corridors characterized by suburban and rural environments, 700 MHz coverage provides essential service enablement that higher frequencies cannot match despite superior theoretical capacity. The strategic error lies in optimizing for peak capacity (favoring 3.5 GHz) rather than coverage reliability (favoring 700 MHz), when railway safety-critical applications prioritize service availability over throughput.

The cell size advantage of low-band deployments, the 82 km maximum radius observed for n28 versus the typical 2–5 km for n78, directly addresses the cell complexity degradation mechanism identified in Section 4.1. Fewer, larger cells inherently reduce visible cell count and cell overlap, decreasing AFC tracking instability without requiring parameter optimization or infrastructure modifications. This architectural benefit makes 700 MHz deployment a structural solution addressing root cause, whereas 3.5 GHz densification to achieve equivalent coverage exacerbates the cell complexity problem it attempts to solve.

### 4.5. Degradation Hotspots

**Figure 7:**
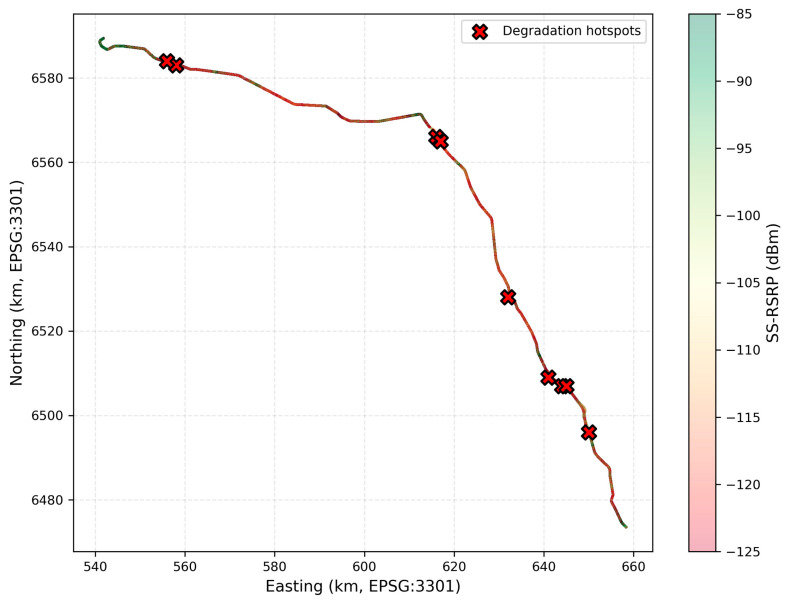
Geographic distribution of identified degradation hotspots along the railway corridor. Background points colored by SS-RSRP signal quality (green = good, red = poor). Nine hotspots marked with red X symbols exhibit mean RSRP below −110 dBm (5.4 to 18.0 dB worse than route median) despite moderate velocities (54–66 km/h mean, zero measurements above 90 km/h). Hotspots correlate with zones of excessive cell overlap (40–115 visible cells) rather than high-speed segments.

The identification of nine discrete geographic hotspots exhibiting disproportionate degradation (Table 4) enables targeted mitigation strategies that address 12.5 dB worst-case degradation through infrastructure modifications rather than requiring system-wide parameter changes. These hotspots, spanning only 9–15 km of the total 185 km corridor (Figure 7) (approximately 5–8% of route length), account for disproportionate poor-quality exposure due to trains spending substantial time traversing these problematic segments at operational speeds.

Geographic analysis reveals that hotspots correspond to zones where coverage from multiple network operators overlaps extensively without coordination. Hotspot HS-4 (115 visible cells, −115.4 dBm mean RSRP) occurs at kilometer 89.3 where urban coverage from Tallinn-region base stations, Tapa town infrastructure, and long-range rural cells converge, creating excessive cell complexity despite moderate 62.1 km/h mean velocity. Similar patterns manifest at HS-1 (87 visible cells, −122.3 dBm) and HS-7 (101 visible cells, −119.5 dBm), suggesting that cell overlap management represents the critical intervention point.

Mitigation strategies for identified hotspots include selective cell deactivation (turning off distant low-RSRP cells contributing to complexity without providing handover benefit), antenna downtilt optimization (reducing coverage extent of base stations beyond their intended service areas), and coordinated network planning among operators (establishing interference coordination zones near railway corridors). These infrastructure-focused approaches address root cause (excessive visible cell count) rather than attempting to compensate for symptoms through user equipment enhancements.

The geographic specificity of hotspot locations (precise kilometer positions identified in Table 4) enables cost-effective targeted deployment. Rather than corridor-wide infrastructure upgrades costing millions of euros, operators can focus on 9–15 km of the total route requiring intervention, potentially reducing mitigation costs by a factor of 10–20 compared to undirected network densification approaches. The return on investment for hotspot-targeted improvements appears favorable given that these locations disproportionately impact user experience despite representing a small fraction of the total route length.

### 4.6. Comparison with International Railway 5G Studies

Recent empirical measurements from Taipei Metro established that 96% of downlink packet loss occurs during handover-related intervals [11], encompassing Master Cell Group Failure, Radio Link Failure, and handover interruption events. Our findings complement this observation: while handover events create episodic disruption, as demonstrated by Chen et al., we identify that continuous within-cell degradation under high cell complexity accounts for 60% of total degradation, operating independently of handover frequency. The distinction proves critical: handover events cause disruption (as Taiwan measurements demonstrate), but cell complexity creates persistent degradation throughout cell residence.

Chinese high-speed railway field measurements at 350 km/h recommend hysteresis margin reductions to 1.84 dB [12] for optimal handover performance at extreme velocities. Our findings at more typical operational speeds (80–120 km/h) indicate threshold adjustments from the observed −115 dBm to the recommended −105 dBm, combined with TTT reduction to 160–256 milliseconds, addresses the late triggering identified in Section 3.5. The geographic and velocity context differences between Chinese high-speed rail-dedicated infrastructure versus Estonian mixed-traffic conventional rail suggest that handover parameter optimization must account for infrastructure type, with conventional railways requiring earlier triggering to compensate for less ideal radio environment.

European 5GRAIL field trials on German and French railways validated 5G standalone performance at the 900 MHz and 1900 MHz FRMCS-designated bands, demonstrating successful voice, emergency calls, and ETCS signaling [13,30]. Spanish railway validation established −120 dBm RSRP threshold sufficient for 99% availability [31] in signaling applications, though this threshold represents minimum acceptable level rather than optimal quality. Our finding that handovers are triggered at a mean of −115.5 dBm positions Estonian networks near this minimum threshold, operating with minimal margin for unexpected degradation events. The Spanish success at −120 dBm confirms feasibility but does not indicate optimality: our analysis suggests that earlier handover triggering would improve quality without compromising availability.

The SOAR Beijing–Shanghai dataset (1788 GB covering 135,719 km) [32] focused on throughput characterization rather than degradation mechanism isolation, representing complementary rather than competing research directions. Our 370 km corridor with 13.7 million measurements provides 100-fold greater measurement density per kilometer (37,000 measurements/km versus 100 measurements/km in SOAR), enabling fine-grained geographic hotspot identification impossible in throughput-focused sampling. The methodological difference highlights tradeoff between geographic coverage extent and measurement granularity, with our approach optimizing for mechanistic understanding over operational area breadth.

Finnish nationwide measurements across 6000 km validated public MNO network capability [15] for FRMCS requirements, demonstrating that dedicated railway networks may not be essential for safety-critical applications. Our Estonian findings provide complementary evidence for the Baltic region, establishing that with appropriate parameter optimization and frequency band allocation, existing commercial 5G infrastructure can meet railway communication requirements. The Nordic-Baltic research synergy positions the region as European leader in practical FRMCS validation, contrasting with Central European emphasis on dedicated infrastructure approaches.

### 4.7. Implications for Future Railway Mobile Communication System

The paradigm shift from velocity-centric to cell-complexity-centric degradation attribution alters FRMCS implementation priorities and success criteria. Traditional approaches emphasizing user equipment Doppler compensation sophistication and high-bandwidth 3.5 GHz capacity allocation emerge as addressing secondary factors while overlooking primary degradation mechanisms. Optimization focus must redirect toward network planning approaches: cell overlap minimization, 700 MHz coverage prioritization, and geographic hotspot targeting. This contrasts with the approaches on other transport modes, such as maritime transport, where the solution is increasing the saturation of base stations [33].

Current European FRMCS spectrum allocations emphasize 1900 MHz (uplink: 1900–1910 MHz) and 900 MHz (downlink: 874–880/919–925 MHz) as primary bands [1,26], with 3.5 GHz designated for capacity supplementation in high-traffic corridors. Our findings validate the prioritization of 900 MHz but suggest more aggressive 700 MHz integration than currently planned, given the 19 dB performance advantage and 98.1% geographic superiority demonstrated in Section 3.2. The 700 MHz band’s status as commercial mobile broadband spectrum rather than railway-dedicated allocation necessitates regulatory coordination, though public–private spectrum sharing models (as pioneered in Finland) provide a precedent.

The cell complexity degradation mechanism identified in Section 4.1 implies that railway corridor radio environment management constitutes critical FRMCS success factor beyond traditional frequency planning. Establishing “railway radio corridors” with regulated cell overlap limits, coordinated antenna patterns among operators, and mandatory interference mitigation zones represents a novel network planning approach not currently addressed in FRMCS specifications. Such regulations parallel railway signaling system protection zones that restrict electromagnetic interference sources near tracks, extending the concept to radiofrequency interference from excessive cell overlap.

Handover parameter optimization recommendations (Section 4.3) align well with FRMCS latency requirements, as earlier handover triggering at −105 dBm reduces time spent in poor-quality states that increase Radio Link Failure probability. The 10 dB improvement in the handover trigger threshold (from the observed −115 dBm to the recommended −105 dBm) translates to an approximately 4-fold increase in the effective signal power margin, providing a resilience buffer against fast fading and interference transients that could otherwise cause communication interruption during safety-critical operations.

The geographic hotspot mitigation strategy (Section 4.5) enables risk-based FRMCS deployment where infrastructure investments concentrate on problematic segments identified through measurement campaigns rather than uniform corridor treatment. This targeted approach aligns with the railway safety management principles of hazard identification and risk mitigation, applying a telecommunications infrastructure planning methodology consistent with broader railway operational frameworks. The 5–8% route length requiring targeted intervention suggests that comprehensive FRMCS quality assurance may be achievable at a fraction of the costs assumed in uniform densification scenarios.

### 4.8. Study Limitations and Methodological Considerations

This study’s single-day measurement campaign took place on 19 November 2025, limiting temporal generalizability, as seasonal effects (particularly winter snow/ice impacts on propagation), diurnal traffic loading variations, and episodic interference sources remain uncharacterized. Specifically, summer foliage density introduces additional vegetation attenuation—typically 3–8 dB for sub-6 GHz frequencies depending on tree species and depth (ITU-R P.833)—which would disproportionately affect n78 (3.5 GHz) relative to n28 (700 MHz) given the frequency-dependent penetration characteristics documented in Section 4.4; summer measurements might therefore show a larger frequency performance gap than the 19 dB observed here. Conversely, the November measurement date represents a favorable seasonal window in one respect: deciduous foliage had largely fallen by mid-November in Estonia, meaning vegetation attenuation was near its annual minimum and the measured signal levels reflected open-path conditions closer to those assumed in standard propagation models. Snow and ice accumulation, which can affect both antenna performance and multipath scattering, were absent on the measurement day (ambient temperature 2–5 °C, dry conditions), though winter precipitation events and their propagation effects remain uncharacterized. The 6 h 8 min measurement window captured typical weekday operations but did not span peak/off-peak transitions or weekend traffic patterns that might alter network loading and handover behavior. Future longitudinal studies tracking performance across weeks or months would strengthen conclusions regarding persistent versus transient degradation patterns.

Geographic scope restriction to the Tallinn–Tartu corridor, while providing detailed single-route characterization, limits generalizability to other Estonian routes and European railway corridors with different infrastructure characteristics. The corridor’s mix of urban, suburban, and rural environments provides representative diversity, though the findings should be applied to high-speed rail (HSR) scenarios exceeding 200–300 km/h with caution. At 350 km/h on the n78 band (3.5 GHz), the Doppler shift reaches approximately ±1134 Hz—representing 3.8% of the 30 kHz subcarrier spacing—compared to only 1.3% at the 120 km/h maximum observed in this study. This nearly threefold increase in relative frequency offset approaches the regime where Doppler-induced inter-carrier interference begins to materially degrade OFDM demodulation performance, and where the velocity-centric degradation paradigm challenged here may regain explanatory relevance. Chinese high-speed rail measurements at 350 km/h [12] demonstrating hysteresis margin dependencies are consistent with this interpretation. The present findings are therefore explicitly geared towards low-to-moderate speed conventional railways (up to ~135 km/h), and independent validation on dedicated HSR infrastructure remains an important future research direction. Pure urban metro environments, characterized by dense cell topologies and frequent stops, may similarly exhibit different relative contributions from cell complexity and velocity effects. The absence of tunnels on this route leaves tunnel-specific propagation challenges unaddressed, though tunnel radio environments typically simplify to a two-ray propagation model with reduced complexity compared to open-air multi-cell scenarios.

The measurement equipment configuration monitoring 10 channels captures substantial, but not comprehensive, network activity, as Estonian operators deploy additional 5G bands beyond those selected for monitoring. The focus on specific ARFCNs (particularly n78 and n28 bands) provides deep characterization of primary bands but may miss performance patterns on secondary or load-balancing channels. Future studies with expanded channel monitoring or multi-equipment deployments could validate whether unmonitored frequencies exhibit similar cell complexity patterns or offer superior performance through reduced inter-operator interference.

The velocity measurement accuracy, derived from GPS position deltas between consecutive samples, introduces uncertainty in velocity-degradation correlation analysis, particularly during acceleration/deceleration zones where velocity changes rapidly within measurement intervals. GPS positioning accuracy of 2–5 m translates to a velocity uncertainty of ±3–7 km/h at typical 200-millisecond sampling intervals, potentially obscuring fine-grained velocity-dependent effects. Integration with train control system velocity telemetry would improve accuracy, though commercial railway operators typically restrict access to such operational data for security reasons.

The study’s focus on downlink signal quality (SS-RSRP, SS-SINR) characterizes user equipment reception but does not capture uplink performance asymmetries that might emerge from different transmit power budgets and antenna configurations. This focsdownlink focus is a hardware constraint of the passive scanning methodology: the ROMES2 scanner operates as a receive-only device, capturing all detectable downlink reference signals simultaneously across multiple operators without requiring active network registration, which is precisely what enables the multi-channel, multi-member measurement architecture central to this study. Uplink characterization requires either active UE-based test equipment with operator cooperation or network-side probe data, neither of which was available for this campaign. It is also worth noting that the DL–UL link budget is not symmetric in 5G NR: uplink power control allows the UE to increase transmit power as path loss increases, partially compensating for the same coverage gaps that produce poor downlink RSRP. Whether this compensation is sufficient to maintain FRMCS uplink reliability in the nine identified hotspots—where downlink RSRP reaches −122 dBm—is an open question that the present data cannot answer, but which represents the most operationally critical gap for future measurement campaigns. Railway operational communications, particularly ETCS data messages and emergency calls, depend on reliable uplink connectivity, suggesting that complementary uplink-focused measurements would provide a complete FRMCS readiness assessment. The assumption that downlink degradation patterns reflect uplink behavior requires validation through dedicated uplink measurement campaigns.

## 5. Conclusions

This study presents empirical evidence in railway 5G degradation research. Analysis of 13.7 million measurements across 370 km of Estonian railway corridor reveals that cell complexity, not velocity, constitutes the primary driver of signal quality degradation in operational 5G networks. The identification of nine geographic hotspots exhibiting 5.4 to 18.0 dB degradation at moderate velocities (54–66 km/h mean, zero measurements above 90 km/h) directly contradicts classical Doppler theory predictions of maximum degradation at the highest speeds.

The quantitative evidence establishes cell complexity as a 3.25 times stronger predictor of signal degradation than velocity (r = −0.390 versus r = −0.120), with visible cell counts of 40 to 115 cells at hotspot locations. We propose that this degradation pattern is consistent with automatic frequency control tracking instability arising from high cell ID churn rates: when serving cell identity changes every 200–500 milliseconds, AFC convergence time may be insufficient before the next transition, producing inter-carrier interference during transient periods. This hypothesis represents a fundamentally different degradation mechanism than velocity-proportional Doppler frequency offsets that modern 5G systems compensate effectively once AFC converges and is supported by the spatial and statistical patterns in our data, though direct confirmation requires measurement of internal modem frequency-lock state variables.

The degradation mechanism decomposition reveals within-cell continuous effects contribute 60% of the total degradation (7.85 dB from low to high speed), while handover boundary effects contribute 40% (2–6 dB additional degradation near cell transitions). This attribution clarifies that both cell complexity and handover timing (manifesting as boundary-specific quality drops) operate simultaneously, with cell complexity dominating the total degradation budget. The finding that handover execution improves signal by +0.30 dB on average, despite near-handover measurements showing worse quality, indicates that the problem lies in late triggering (mean −115.5 dBm) rather than handover mechanism failure.

Frequency band analysis demonstrates overwhelming 700 MHz (n28) superiority at 98.1% of corridor locations with 19 dB performance advantage over 3.5 GHz (n78), validating low-frequency propagation physics over capacity-focused higher-frequency approaches for railway environments. The finding that only 3.4 km of the 185 km corridor favored 3.5 GHz, concentrated in dense urban cores, challenges current European FRMCS spectrum strategies prioritizing 3.5 GHz capacity while treating 700 MHz as a supplementary coverage band. For railway applications requiring reliable connectivity across diverse environments, coverage trumps capacity.

The paradigm shift from velocity-centric to cell-complexity-centric degradation attribution redirects optimization priorities from user equipment Doppler compensation algorithms toward network planning approaches: cell overlap minimization through selective deactivation and antenna optimization, 700 MHz prioritization over 3.5 GHz for primary coverage, handover parameter adjustment enabling earlier triggering at −105 dBm rather than −115 dBm, and geographic hotspot targeting concentrating infrastructure investment on 9–15 km problematic segments rather than uniform corridor treatment.

As the first comprehensive empirical 5G measurement study in the Baltic region, this research addresses a critical knowledge gap in European railway communications research. While Nordic countries have conducted measurement campaigns (Finland’s 6000 km nationwide study) and Estonian planning simulations exist, no prior work has provided detailed signal quality characterization for Baltic railway corridors despite Estonia’s coordinator role in Rail Baltica 5G infrastructure planning. The dataset’s scale—13.7 million measurements, representing a 100-fold greater density per kilometer than typical throughput-focused studies—enables geographic hotspot identification and mechanism isolation impossible with sparse sampling.

The practical implications extend beyond immediate Estonian network optimization to influence broader FRMCS implementation strategy across Europe. The cell complexity mechanism suggests that railway radio corridor protection zones, analogous to airport approach path electromagnetic environment management, may prove necessary to prevent excessive cell overlap from uncoordinated operator deployments. The 700 MHz performance advantage, though expected from propagation theory, receives quantitative empirical validation (19 dB, 98.1% geographic superiority) that strengthens arguments for regulatory spectrum allocation prioritizing railway-optimized frequency bands.

The study limitations—single-day temporal scope, single-corridor geographic coverage, and downlink-focused measurement—suggest clear directions for future research. Multi-season campaigns capturing winter propagation effects and summer vegetation attenuation, expanded geographic coverage across Estonian railway network and Rail Baltica corridors into Latvia and Lithuania, and uplink performance characterization complementing the current downlink focus would strengthen conclusions and validate generalizability. Coordinated measurements with user equipment manufacturers accessing internal AFC state variables would provide definitive validation of the proposed tracking loop mechanism.

The convergence of empirical evidence from multiple sources—Estonian measurements showing cell complexity dominance, Taiwanese metro data [11] demonstrating 96% handover-related packet loss, Chinese high-speed rail [12] validating parameter optimization at extreme velocities, and European FRMCS trials [13,30] confirming public network feasibility—establishes robust international understanding of railway 5G performance characteristics. Each study contributes complementary perspectives: Taiwan characterizes handover disruption at the macro level, China optimizes extreme-velocity scenarios on dedicated infrastructure, Europe validates safety-critical application feasibility, and Estonia isolates degradation mechanisms through fine-grained geographic analysis.

The research contributions can be summarized as follows. First, we provide an empirical demonstration that cell complexity, not velocity, dominates railway 5G signal degradation, with a 3.25-fold stronger correlation and 0% high-speed measurements in the worst hotspots, contradicting the Doppler-centric paradigm. Second, we provide a proposed mechanistic explanation—consistent with AFC tracking instability under high cell ID churn rates—that is distinct from classical Doppler frequency offset compensation and is supported by the observed spatial correlation between cell density and degradation, pending direct validation through modem baseband state measurement. Third, we perform quantitative degradation decomposition, separating within-cell continuous effects (60%, 7.85 dB) from handover boundary effects (40%, 2–6 dB), enabling targeted mitigation strategies addressing each component independently.

Fourth, we perform frequency band performance quantification, establishing the superiority of 700 MHz (19 dB advantage, 98.1% geographic dominance) with propagation physics implications for FRMCS spectrum allocation strategies. Fifth, we perform geographic hotspot identification, providing precise locations (nine hotspots at identified kilometer positions) for targeted infrastructure investment, achieving 10–20× cost efficiency compared to corridor-wide improvements. Sixth, we perform handover parameter analysis, revealing late triggering at a mean of −115.5 dBm despite a +0.30 dB improvement from handover execution, indicating timing rather than mechanism problems.

The findings position network planning as a higher-leverage optimization domain than user equipment enhancement for railway 5G performance improvement. While user equipment Doppler compensation improvements offer marginal benefits at already-managed ±389 Hz frequency offsets (at 120 km/h, 3.5 GHz), network-side interventions addressing excessive cell overlap, frequency band misallocation, and handover timing tackle root causes of 12.5 dB worst-case degradation observed in operational networks. This prioritization proves particularly relevant for FRMCS, where infrastructure longevity (15–20 year operational lifetime) amplifies the importance of correct initial deployment decisions.

Future research directions include validation of cell complexity mechanisms through direct frequency offset measurements during cell transitions, expansion to additional European railway corridors assessing generalizability across infrastructure types and operational patterns, integration with train control system data enabling joint optimization of railway operations and telecommunications performance, and development of predictive models forecasting degradation likelihood from network topology parameters, enabling proactive intervention before hotspot formation.

The broader impact extends to mobile network planning beyond railway applications, as the cell complexity degradation mechanism likely affects other high-mobility scenarios, including highways, airports, and urban transit systems. The finding that 40–115 visible cells create persistent tracking failure suggests that network densification strategies, while beneficial for capacity, risk creating performance degradation zones unless accompanied by careful overlap management. This insight challenges conventional assumptions that more cells universally improve service quality, revealing complexity threshold beyond which additional cells degrade rather than enhance performance.

For further research based on analyzed data insights targeting network improvement we see the need for the simulation of beamforming scenarios and overall radio and capacity simulation when using time temporal targeted antenna use. This is required for the creation of cost–benefit model ideas on revenue (e.g., premium service), cost saving (e.g., capex investments/opex needed), and strategic benefits. In addition, several potential data inputs are to be evaluated for use in prediction or real-time triggering of optimized antenna usage, like rail transport (train schedules, tickets, maintenance), public transport monitoring, telecom network data, environmental and contextual data (events seasonality, weather models), and industrial and operational data (energy grid, logistics). AI models can combine these data streams to predict real-time network load, optimize beamforming and antenna steering, allocate radio resources, or trigger automated network actions.

In conclusion, this study establishes an empirical foundation for a paradigm shift in railway 5G optimization, redirecting focus from velocity-dependent Doppler effects toward network topology management. The cell complexity mechanism, frequency band physics, and geographic hotspot targeting provide an actionable framework enabling European railways to achieve FRMCS reliability requirements through evidence-based deployment strategies. As commercial 5G networks evolve toward supporting safety-critical railway applications, understanding which degradation mechanisms dominate operational environments, versus simulation environments, proves essential for successful infrastructure investment and service quality assurance.

Future European railway digitalization success depends on rigorous empirical validation of network performance under realistic operational conditions, incorporating the complexity of multi-operator commercial networks, diverse geographic environments, and actual traffic patterns that characterize modern railway corridors. This study provides methodology, findings, and practical recommendations advancing that essential validation process, positioning the Baltic region as a contributor to the European FRMCS knowledge base while addressing immediate Estonian railway telecommunications optimization needs.

## Figures and Tables

**Table 1:** Velocity distribution across measurement campaign showing sample sizes and percentage representation in each speed category.

Velocity Range	Measurements	Percentage	Mean Velocity
0–30 km/h	3,014,132	22.00%	14.8 km/h
30–60 km/h	1,768,038	12.90%	45.2 km/h
60–90 km/h	2,311,658	16.90%	76.1 km/h
90–120 km/h	5,257,739	38.30%	104.7 km/h
120–150 km/h	1,358,631	9.90%	132.4 km/h
Total	13,710,327	100%	75.3 km

**Table 2:** Geographic frequency band performance comparison across railway corridor.

Band	Frequency	Superior Locations	Route Length	Mean RSRP	RSRP Advantage
n28	700 MHz	98.10%	181.7 km	−102.3 dBm	Baseline
n78	3.5 GHz	1.90%	3.4 km	−121.3 dBm	−19.0 dB
n3	1800 MHz	0%	0 km	−117.5 dBm	−15.2 dB

**Table 3:** Signal quality metrics stratified by velocity bins showing mean values and degradation relative to baseline low-speed conditions.

Velocity Bin	SS-RSRP (dBm)	Δ from 0 to 30	SS-SINR (dB)	Δ from 0 to 30	Sample Size
0–30 km/h	−104.2	Baseline	−9.8	Baseline	934,889
30–60 km/h	−104.4	−0.2 dB	−10.9	−1.1 dB	486,751
60–90 km/h	−110	−5.8 dB	−12.5	−2.7 dB	750,411
90–120 km/h	−112.3	−8.1 dB	−12.6	−2.8 dB	1,684,258
120–150 km/h	−112.2	−8.0 dB	−12.6	−2.8 dB	379,465

**Table 4:** Characteristics of nine identified signal quality hotspots showing moderate velocities and extreme degradation.

Hotspot ID	Location (km)	Mean RSRP	Degradation	Mean Velocity	Max Velocity	Cell Count
HS-1	23.4	−122.3 dBm	−18.0 dB	66.4 km/h	88 km/h	87
HS-2	45.7	−118.9 dBm	−14.6 dB	54.3 km/h	78 km/h	62
HS-3	67.2	−116.8 dBm	−12.5 dB	58.7 km/h	83 km/h	73
HS-4	89.3	−115.4 dBm	−11.1 dB	62.1 km/h	87 km/h	115
HS-5	112.8	−114.2 dBm	−9.9 dB	59.8 km/h	81 km/h	94
HS-6	134.5	−113.7 dBm	−9.4 dB	61.3 km/h	86 km/h	68
HS-7	156.1	−119.5 dBm	−15.2 dB	55.9 km/h	79 km/h	101
HS-8	168.9	−115.9 dBm	−11.6 dB	63.4 km/h	89 km/h	58
HS-9	179.2	−114.6 dBm	−10.3 dB	57.6 km/h	84 km/h	77
Mean	-	−116.8 dBm	−12.5 dB	60.2 km/h	84 km/h	82 cells

**Table 5:** Correlation analysis comparing velocity and cell complexity as predictors of signal quality degradation.

Predictor Variable	Pearson r	*p*-Value	R^2^	Interpretation	Relative Strength
Velocity (km/h)	−0.12	<0.001	0.014	Very weak	Baseline (1.0×)
Cell Complexity (count)	−0.39	<0.001	0.152	Moderate	3.25× stronger
Velocity (hotspots only)	−0.089	31.20%	0.008	Non-significant	0.74×
Cell Complexity (hotspots only)	−0.547	<0.001	0.299	Moderate-Strong	4.56× stronger

**Table 6:** Degradation decomposition separating within-cell continuous effects from handover boundary effects across velocity bins.

Velocity Bin	Within-Cell RSRP	Near-Handover RSRP	Boundary Degradation	Sample Sizes (Within/Near)
0–30 km/h	−103.7 dBm	−109.8 dBm	−6.1 dB	870,188/73,323
30–60 km/h	−104.0 dBm	−106.8 dBm	−2.8 dB	478,830/83,911
60–90 km/h	−109.7 dBm	−111.7 dBm	−2.0 dB	618,660/123,929
90–120 km/h	−111.6 dBm	−115.8 dBm	−4.2 dB	1,395,223/289,035
120–150 km/h	−111.4 dBm	−116.2 dBm	−4.8 dB	364,627/70,995

**Table 7:** Handover signal quality transitions showing distribution of improvements, degradations, and neutral outcomes.

Handover Outcome Category	RSRP Change Range	Event Count	Percentage	Mean RSRP Change
Significant Improvement	>+3 dB	165,877	0.316	+8.2 dB
Neutral Transition	−3 to +3 dB	211,021	0.402	+0.1 dB
Significant Degradation	<−3 dB	148,028	0.282	−7.9 dB
Overall	All ranges	524,926	1	+0.30 dB

## Data Availability

The raw data supporting the conclusions of this article will be made available by the authors on request.

## Abbreviations

The following abbreviations are used in this manuscript:

5G/5G NR	Fifth-generation mobile network/New Radio (5G air interface)
ARFCN	Absolute Radio Frequency Channel Number
PCI	Physical Cell ID
SSB	Synchronization Signal Block
SSB-RSSI	SSB Received Signal Strength Indicator
SS-RSRP	SS Reference Signal Received Power
SS-RSRQ	SS Reference Signal Received Quality
SS-SINR	SS Signal-to-Interference-plus-Noise Ratio
AFC	Automatic frequency control
OFDM	Orthogonal Frequency-Division Multiplexing
FR1/FR2	5G NR Frequency Range 1 (sub-6 GHz)/Range 2 (mmWave)
n28/n3/n78	5G NR bands: 700 MHz/1800 MHz/3.5 GHz
n100/n101	FRMCS railway bands: 900 MHz/1900 MHz
FRMCS	Future Railway Mobile Communication System
GSM-R	Global System for Mobile Communications–Railway
ETCS	European Train Control System
RRC	Radio Resource Control
RACH	Random Access Channel
A3 event	Handover trigger condition (neighbor becomes better)
TTT	Time to Trigger (handover delay parameter)
RLF	Radio Link Failure
FSPL	Free-space path loss
GPS	Global Positioning System
ROMES2	Rohde & Schwarz 5G NR measurement/scanner platform
PostGIS	Geospatial extension for PostgreSQL
EPSG:3301	Estonian National Grid coordinate system
DBSCAN	Density-Based Spatial Clustering of Applications with Noise
GIST	Generalized Search Tree (spatial index)
MNO	Mobile Network Operator
5GRAIL/5G REMORA/5G-RACOM	European railway 5G research projects
SOAR	Beijing–Shanghai railway measurement dataset
r/R2	Pearson correlation coefficient/variance explained
*p*-value	Statistical significance indicator
Cohen’s d	Effect size measure
